# Craniomaxillofacial Trauma in Dogs—Part I: Fracture Location, Morphology and Etiology

**DOI:** 10.3389/fvets.2020.00241

**Published:** 2020-04-28

**Authors:** Mercedes H. De Paolo, Boaz Arzi, Rachel E. Pollard, Philip H. Kass, Frank J. M. Verstraete

**Affiliations:** ^1^School of Veterinary Medicine, William R. Pritchard Veterinary Medical Teaching Hospital, University of California, Davis, Davis, CA, United States; ^2^Department of Surgical and Radiological Sciences, University of California, Davis, Davis, CA, United States; ^3^Department of Population Health and Reproduction, School of Veterinary Medicine, University of California, Davis, Davis, CA, United States

**Keywords:** craniomaxillofacial, trauma, computed tomography, fracture, displacement, dog

## Abstract

Treatment of craniomaxillofacial (CMF) trauma in dogs often requires a multidisciplinary approach and a thorough understanding of the CMF skeletal structures involved. The aim of this retrospective study was to use a large number of CT studies of dogs evaluated for CMF trauma and to describe fracture location and morphology in relation to demographic data and trauma etiology. The medical records and CT studies of 165 dogs over a 10-year period were evaluated. The skeletal location of CMF fractures as well as the severity of displacement and fragmentation of each fracture was recorded. Patient demographic data and trauma etiology were also recorded. Animal bites accounted for the majority of trauma (50%), followed by unknown trauma (15%), vehicular accidents (13%), and blunt force trauma (13%). Small dogs, <10 kg, and juveniles accounted for the majority of patients (41.8 and 25.5%, respectively). The most likely bone or region to be fractured was the maxillary bone, followed by the premolar and molar regions of the mandible. Up to 37 bones or regions were fractured in any given patient, with an average of 8.2 fractured bones or regions per dog. The most commonly fractured location varied according to trauma etiology. Specifically, vehicular accidents tended to result in more locations with a higher probability of fracture than other trauma types. A major conclusion from this study is that every bone of the CMF region was fractured in at least one case and many cases had a large number of fractured regions. Thus, the need for comprehensive assessment of the entire CMF region, preferably using CT, is underscored.

## Introduction

Craniomaxillofacial (CMF) trauma is a relatively common reason for which dogs are presented to veterinarians on an emergency basis. CMF trauma may occur in isolation but often accompanies injury to other parts of the body and therefore requires a multidisciplinary approach to optimize patient care (1–3). Fracture morphology and spatial location play an essential role in clinical decision-making (3, 4). In the human medical literature, several classification systems and injury severity scores for the CMF region have been made (2, 5–8). Perhaps the most well-known of these, the Le Fort fractures, are based on the repeatable lines of weakness of the midface demonstrated by Rene Le Fort in his classic cadaveric studies (9). A multitude of other classification systems and severity scores have been created for people with a recent effort by the professional association AOCMF (Arbeitsgemeinschaft fur Osteosynthesefragen- craniomaxillofacial) to create one standardized, accepted classification system for the entire CMF region (10). At present, there is not a standardized, accepted classification system for the CMF region in dogs.

The potential of such systems lies in their ability to aid in clear and standardized communication between clinicians as well as to provide therapeutic and prognostic information (10, 11). This is especially important in the CMF region as specialists with distinct but overlapping training can be involved in CMF trauma management. For example, in the veterinary field, treatment of CMF trauma patients could conceivably be performed by veterinary dentists, surgeons, neurosurgeons, ophthalmologists, criticalists, and general practitioners. In addition, classification systems may provide a logical explanation for approaches to management of the CMF trauma patient for those practitioners who have less experience in this region (11). The basis of any classification system first requires knowledge of common fracture location and morphology, ideally based upon a large number of cases.

Given the anatomically complex and overlapping nature of structures in the CMF region, it is not surprising that the diagnostic yield of CT in reference to identifying fractures is greater than that of skull radiographs (12, 13). When assessing people who have sustained CMF trauma, CT is considered the standard of care, and there is increasing recognition that three-dimensional and multiplanar reconstructions are extremely important for accurate diagnosis and optimal treatment planning (14). While utilizing the two dimensional aspects of CT is essential for the smaller and more internal CMF fractures, it is well-recognized that the two-dimensional and three-dimensional modalities are best utilized together (9, 15, 16). In some cases, not only is preoperative CT used for diagnostic and planning purposes, but intraoperative CT is also being utilized during surgery and has been shown to change clinical decision-making (17). Fortunately, there is increasing access to CT in veterinary practice, which may improve the accuracy of diagnosis in CMF trauma patients.

In addition to the spatial location of CMF fractures, the fracture morphology also has an influence on treatment planning and outcomes (10). In general, fragmentation and displacement are discussed when describing fracture morphology regardless of the type or location of the fractured bone (18). One aspect of fracture morphology that is different between long bones and bones of the CMF region is that the descriptive terms of linear, spiral, transverse, oblique, etc. are not always applicable in the bones of the CMF region (18). Nevertheless, collecting and describing the severity of displacement and fragmentation of fractures in the CMF region is likely to be useful.

Literature regarding common causes of CMF trauma in dogs is sparse and typically includes animal altercations, vehicular accidents, falls from a height, and unknown trauma as the most common etiologies (19–23). Similarly to trauma etiology, a small number of studies have reported the physical location of fractures secondary to CMF trauma in dogs (19, 21–25). Currently, the mandible is reported to be vastly more likely to be fractured than other parts of the skull (19, 22, 25). In addition, in the mandible, the premolar and molar teeth regions are the most commonly fractured (21), and this may be dependent on patient size (23). Documenting whether these findings are upheld when using computed tomography will provide important information to veterinarians.

To the authors' knowledge, current veterinary literature lacks comprehensive reports detailing CMF fracture location, morphology, etiology, and the relationship of each of these variables in a large number of dogs utilizing CT for diagnosis. This retrospective, descriptive study includes CT findings of 165 dogs that sustained CMF trauma. The primary objectives of the study were: (1) to describe the most common fracture etiology, location, and morphology and (2) to determine whether relevant demographic data (size, age, sex) were related to any of these variables. Although the objective of this study did not include creation of a classification system, the information gathered here can be used for the basis of classification systems in the future. We hypothesized that fracture location and morphology would be influenced by trauma etiology and that demographic variables would influence fracture location, morphology, and trauma etiology. In the accompanying article, entitled “Craniomaxillofacial trauma in dogs- part II,” the associations between fracture location, morphology, and trauma etiology are analyzed further.

## Materials and Methods

### Case Selection

The electronic medical record database of the UC Davis Veterinary Medical Teaching Hospital was searched for dogs that had been presented for evaluation and treatment following CMF trauma between the years 2008 and 2018. For inclusion, all dogs must have undergone CT (conventional CT and/or cone-beam CT [CBCT]) at the initial visit. Exclusion criteria were as follows: trauma that had occurred > 1 week prior to presentation, patients with CT slice thickness of > 1.3 mm, and those for whom either the medical record or CT study were incomplete (e.g., the caudal most portion of the skull had not been included in the images). Cases were excluded if the trauma occurred > 7 days prior to presentation due to concern that (a) early signs of fracture repair and boney remodeling may make fracture identification more difficult and (b) further displacement may have occurred since the trauma. Exclusion of cases if the slice thickness was > 1.3 mm was chosen as a compromise between maximizing the number of cases that were included in the study while simultaneously ensuring that slice thickness was not so large that small or incomplete fractures could be missed.

### Image Acquisition and Evaluation

All dogs underwent conventional CT (HiSpeed FX/i or LightSpeed16, GE Healthcare, Waukesha, WI) and/or CBCT (NewTom 5G CBCT Scanner, NewTom, Verona, Italy) imaging at their initial visit. Although many dogs presenting for CMF trauma at our institution undergo CBCT, including conventional CT allowed the study to capture those patients in which superior soft tissue imaging was medically necessary (e.g., those with concern for intracranial hemorrhage), those too large for the CBCT field of view, and those who received treatment prior to the availability of CBCT at this facility. All DICOM files from each study were viewed using a specialized software (*Invivo*5, Anatomage, San Jose, CA). Each case was viewed dynamically on medical flat-grade monitors (ASUS PB278Q 27-inch, ASUSTeK Computer Inc., Taipei, Taiwan), allowing the observers to utilize all viewing modes and tools to best assess all fractures. One observer (MD) viewed all studies and recorded all data after a period of calibration with one experienced board-certified veterinary radiologist (RP) and two board-certified veterinary dentists and Oral and Maxillofacial Surgery Fellows (FV, BA). When there was uncertainty regarding the presence or severity of a lesion, the study was reviewed with the board-certified radiologist (RP). Although soft tissue injuries were evaluated when the patient was in hospital, they were neither evaluated nor recorded for the purposes of this study.

### Fracture Evaluation

**Figure 1:**
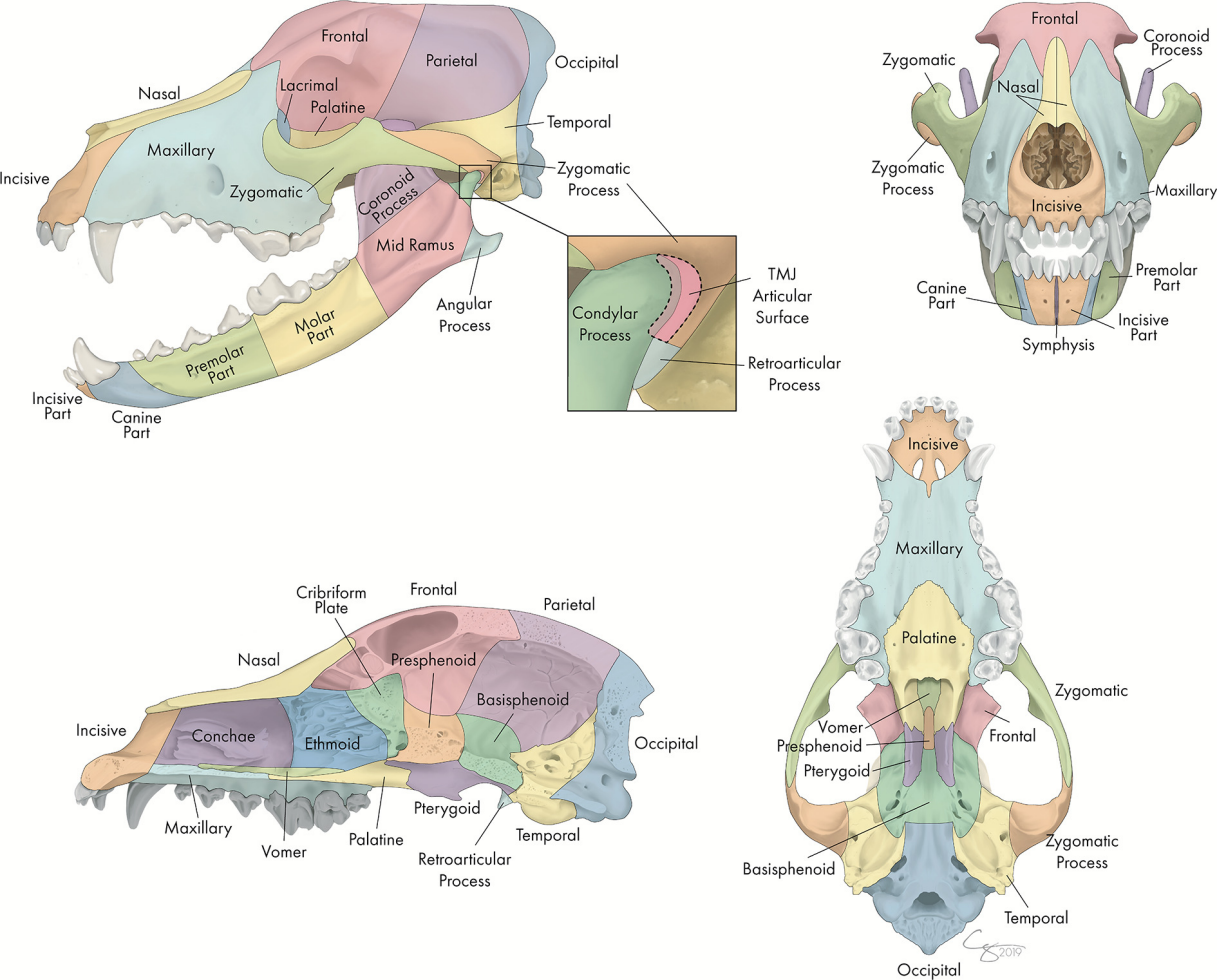
Bones and regions of the CMF skeleton of the dog. This array of skulls was used when assessing which bone and regions of the CMF skeleton were fractured.

Each skull was divided into specific bones and regions as illustrated in Figure 1. For each bone or region, it was determined whether there was a fracture and, if so, fracture morphology was described in terms of displacement and fragmentation. The degrees of displacement and fragmentation were modeled after the AOCMF fracture classification system in humans (10). For both displacement and fragmentation, a score of 0 indicated that there was no fracture. When scoring displacement, a score of 1 indicated that there was no displacement, a score of 2 that there was minimal displacement with < =50% overlap remaining between fragments, and a score of 3 that there was severe displacement with > 50% overlap remaining. When scoring fragmentation, a score of 1 indicated an incomplete fracture, a score of 2 a complete fracture, and a score of 3 was consistent with a comminuted fracture. This process was repeated on both the right and left sides of the skull. Although use of the term “comminuted” is discouraged by the most recent recommendations in human CMF literature (26), the term and its associated meaning are still pervasive in veterinary medicine and was therefore utilized in this study. A comminuted fracture was defined as a fracture having 3 or more bone fragments, although “minute” fragments were ignored unless the entire bone or region had been reduced to microfragments (27).

Because the bones that form the temporomandibular joint (TMJ) may, in themselves, be fractured without there being a fracture that extends into the articular space, fractures of the TMJ were recorded as a unique category separate from the condylar process, the retroarticular process, and the temporal bone. It was expected that there would be frequent overlap between these fractures. However, recording the instances of a fracture involving the articular surface itself was considered important enough to be coded separately. Similarly, although the cribriform plate is technically considered part of the ethmoid bone (28), the possible prognostic implications of having breached the braincase were deemed important enough to record instances of cribriform fracture separately from other ethmoid fractures.

**Figure 2:**
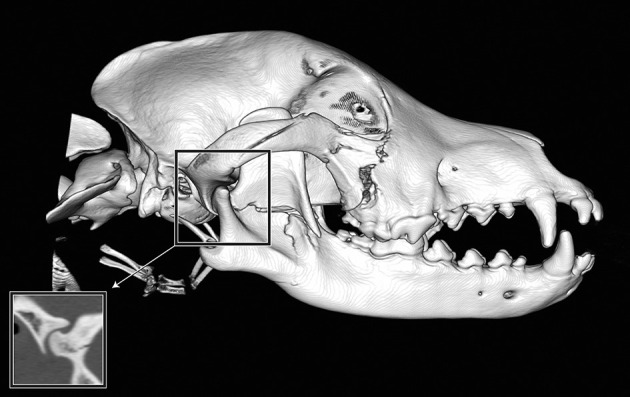
Example of fragmentation and displacement of a single fracture line crossing multiple bones or regions. If the fracture affected individual bones or regions to a greater or lesser extent, the severity was recorded for each bone or region individually. In this example, the fracture line spanned multiple regions of the mandible. The mid-ramus was found to have fragmentation and displacement scores of 2, whereas the condylar process (not including the articular surface) had a fragmentation score of 1 (incomplete fracture) and a displacement score of 1 (no displacement). In addition, there are fractures of the right zygomatic bone.

If a fracture occurred along a suture or at a border between two regions, the bone or region on both sides was considered fractured, and the morphology of the fracture was considered separately for each bone or region. By definition, all fractures along a suture were considered complete. However, the degree of displacement was recorded individually for the bone on either side of a suture (Figure 2).

**Figure 3:**
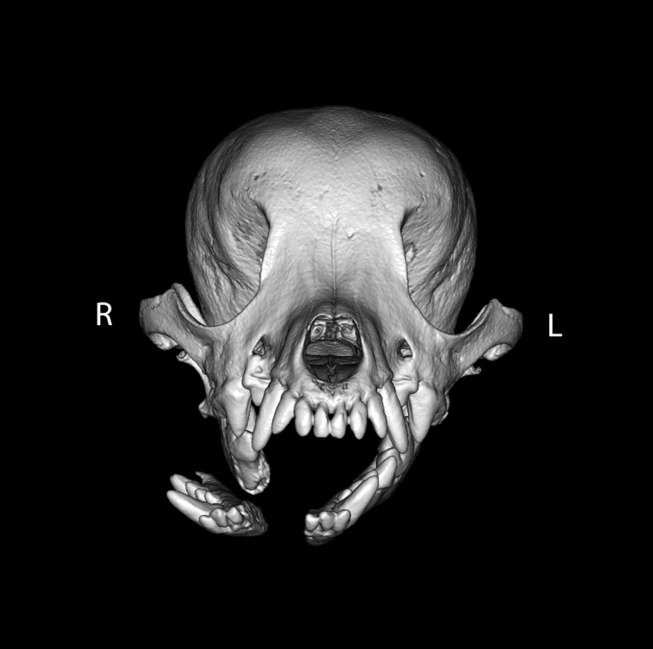
Example of symphyseal separation. By definition, all symphyseal separations were considered to be complete. However, in this example, the two sides of the symphysis were differentially displaced such that the left side of the symphysis remained essentially in place (displacement score of 1), whereas the right side of the symphysis was severely displaced (displacement score of 3).

For the intermandibular joint (symphysis), a fibrocartilaginous joint (synchondrosis), symphyseal separation was considered by definition to be bilateral. However, if the two sides were unequally displaced (Figure 3), the coding reflected this.

### Fracture Etiology

For each case, one of seven different fracture etiologies were assigned, as depicted in Table 1.

**Table 1:** Explanation of fracture etiology codes.

**Fracture etiology**	**Code**	**Examples or clarification**
Crush/slow velocity	1	Slow vehicular roll-over, stepped or sat on, shut in door, etc.
Vehicular injury	2	Any vehicular injury not specified in other categories.
Animal bite	3	Bite originating from any other animal.
Fall from height	4	Fall from building, fall from vehicle, etc.
Ballistic injury	5	Bullet, arrow, etc.
Blunt force trauma	6	Baseball bat, horse kick, running into an object (including a vehicle if vehicle stationary), etc.
Unknown/miscellaneous	7	Unknown injury or not otherwise characterized.

### Demographic Data

Patient sex (male and female, intact or neutered) and age (in years, or portion thereof) were recorded for each case. Although breed and skull shape may be related to fracture location and morphology, for the purposes of this study it was determined that patient weight in kilograms at time of presentation would be the only breed-related variable recorded. Patients were grouped into <10 kg, 10–20 kg, 20–40 kg, and > 40 kg. Additionally, patients who were considered juvenile based on the presence of mixed or deciduous dentition were categorized separately as it was unlikely that their weight at the time of presentation would accurately reflect their final weight.

### Statistical Methods

Exact binomial proportions and confidence intervals were calculated to evaluate the distribution of severe displacement and fragmentation by fracture location. These analyses were also used to assess the proportion of fractures present at each location, conditional on each of four trauma type etiologies for which at least 20 cases were represented in the data.

Fisher's exact test was used to compare trauma etiology across the four distinct groups defined by sex and gonadectomy status. The Kruskal-Wallis test was used to compare age distributions across the same four groups. Fisher's exact tests were also used to compare the association between the categorical variables (sex and size) with fracture location and morphology. Kruskal-Wallis tests were used to compare age between patients with fractures at specific locations and with specific morphologies. In addition, the chi-square test of homogeneity was used to compare the observed and expected counts in contingency tables defined by breed size (weight) categories and fracture etiologies. *P* < 0.05 were considered statistically significant.

## Results

### Descriptive Statistics

**Figure 4:**
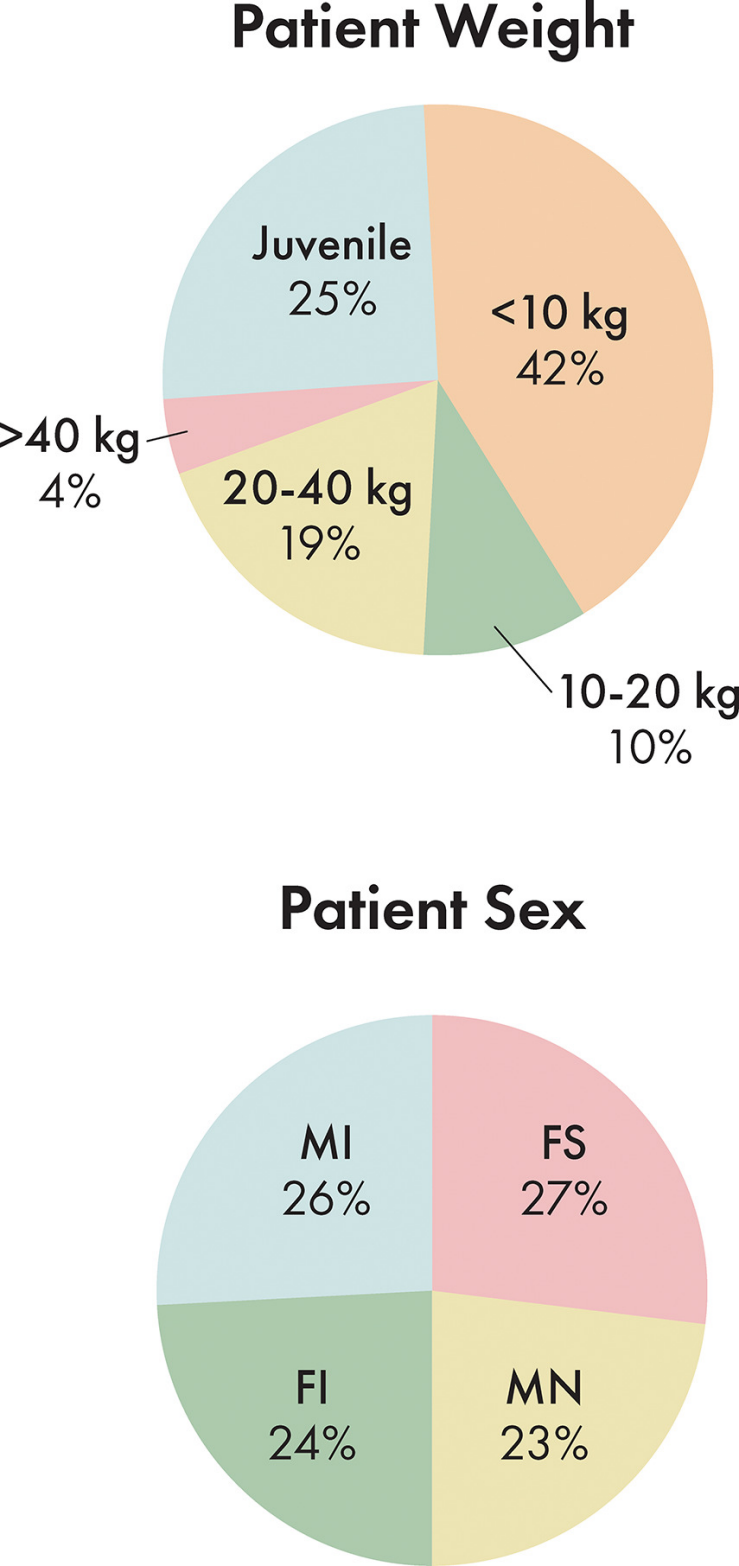
Population distribution by patient size and by patient sex. MI, male intact; FI, female intact; FS, female spayed; MN, male neutered.

Out of 165 dogs evaluated in this study, 45 dogs were spayed females, 39 were intact females, 38 were neutered males, and 43 were intact males (Figure 4). The ages ranged from 2 days to 16 years with an average age of 4.3 years. 25.5% of dogs were considered juvenile based on the dentition. The proportion of dogs in each size bracket was as follows: <10 kg: 41.8%, 10–20 kg: 9.7%, 20–40 kg: 18.8%, and > 40 kg: 4.2% (Figure 4).

**Figure 5:**
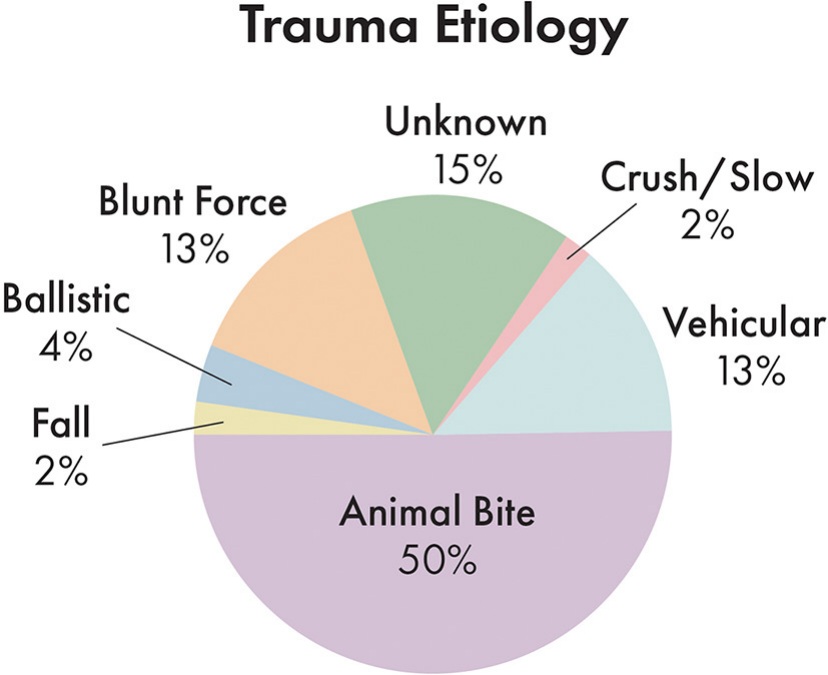
Distribution of trauma etiology. Note that animal bites accounted for the largest proportion of trauma etiologies.

Incidence of trauma etiology, depicted in Figure 5, demonstrated that animal bites caused the majority (50.3%) of injuries. The average number of fractured regions or bones was 8.2 per dog, with up to 37 fractured regions, and only 7.2% of cases (12 dogs) having a solitary fractured region or bone. 41.2% of cases had bilateral fractures for at least one bone or region.

### Most Commonly Fractured Locations

**Figure 6:**
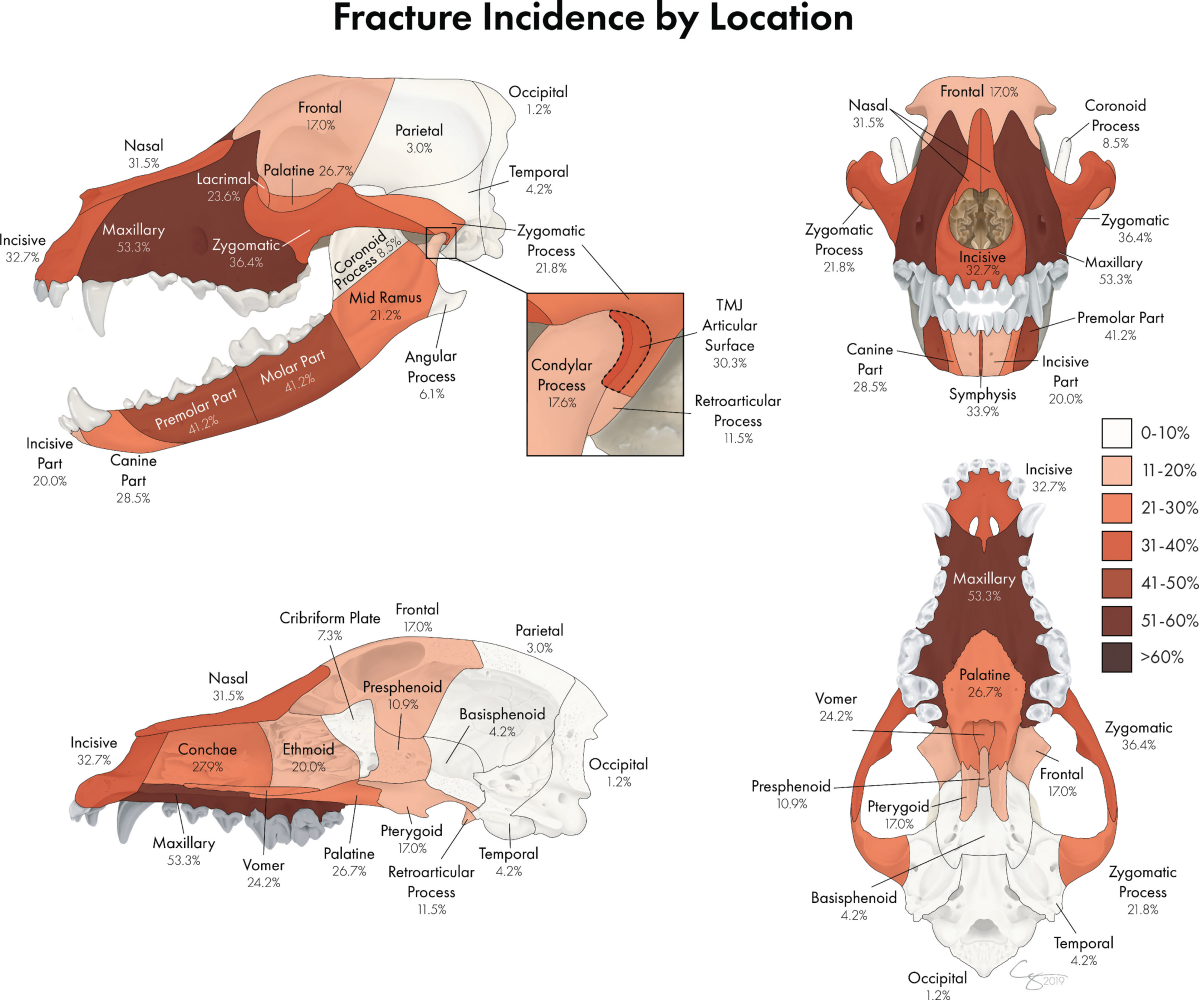
A percentage heat map demonstrating the proportion of CMF fracture locations. Percentages represent the percentage of dogs sustaining a fracture in each location. For example, 53.3% of dogs in this study sustained fractures of the maxillary bone, whereas 1.2% of dogs in this study sustained fractures of the occipital bone.

**Figure 7:**
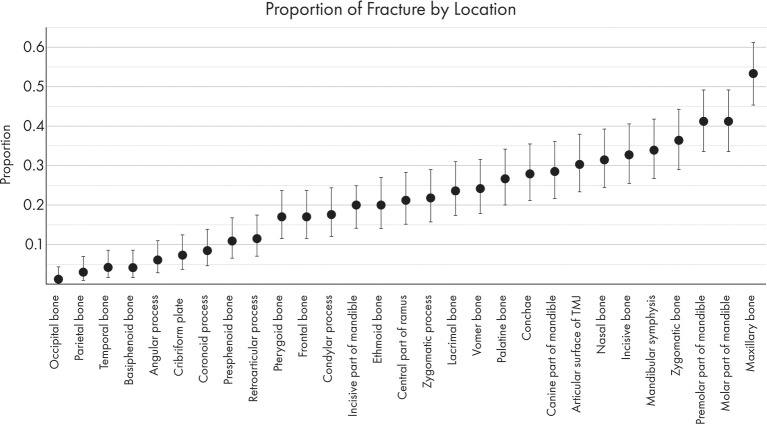
Graphical projection of the proportion of CMF fractures by location. Note that the proportions of fractures spanned the entire CMF region but was the highest at the caudal mandibles and the maxillary bones.

The most commonly fractured location was the maxillary bone (Figures 6, 7), with 53.3% of dogs having sustained at least one fracture of this bone. The molar and premolar parts of the mandible were each affected in 41.2% of dogs. The least commonly affected locations were the occipital and parietal bones with each being fractured in 1.2 and 3% of cases, respectively. There was not a bone or region in the skull that was unaffected in all cases (i.e., no bone/region was fractured in 0% of cases). No attempt was made to determine significance based on possible overlapping of confidence intervals. However, a general trend of increasingly common fractures of the midface (maxilla, zygomatic, nasal, and incisive bones) as well as the premolar and molar parts of the mandible can be seen in Figures 6, 7. The articular surface of the TMJ was fractured in 30.3% of cases.

### Fracture Morphology by Location

**Figure 8:**
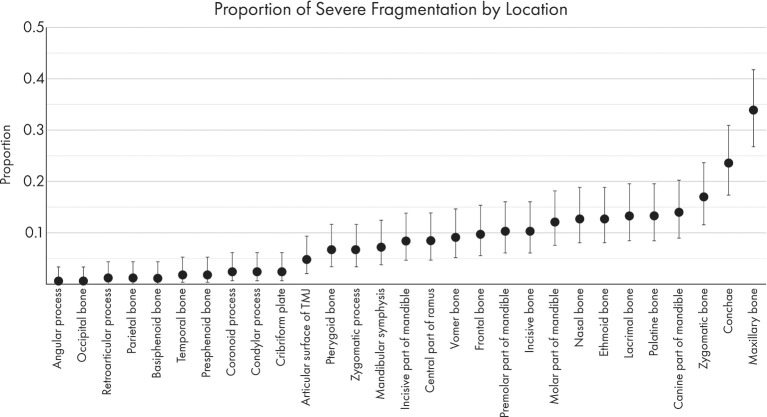
Graphical projection of the proportion of severely fragmented fractures at each location. Note that the proportion of fractures with severe fragmentation (comminution) was highest in the maxillary bone, zygomatic bone, and conchae.

**Figure 9:**
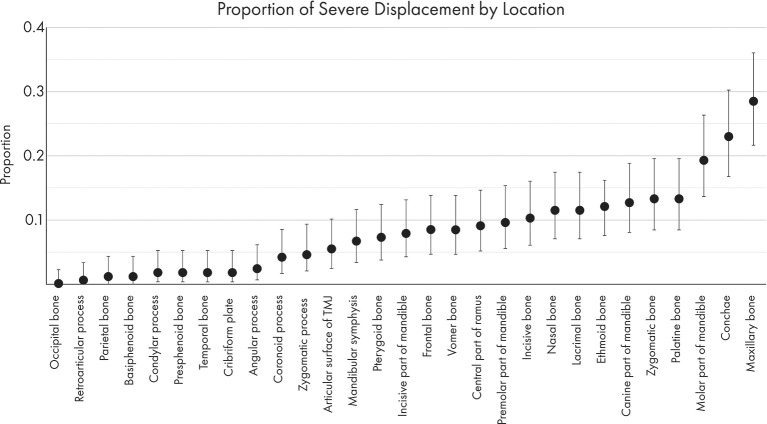
Graphical projection of the proportion of severe displacement at each fracture location. Note that the most severely displaced fractures (<50% overlap between fracture segments) occurred at the maxillary bones, the conchae, and the molar part of the mandible.

The proportions of severely displaced and fragmented fractures in each location are depicted in Figures 8, 9. The maxillary bone had the highest proportion of severely displaced and fragmented fractures, with 28.5 and 33.9% of maxillary fractures being severely displaced or fragmented, respectively. The conchae were affected by severe displacement and fragmentation in ~23% of cases. In general, regions of the mid-face and the body of the mandible were also more likely to be affected by severe fragmentation and displacement.

### Fracture Etiology by Location

**Figure 10:**
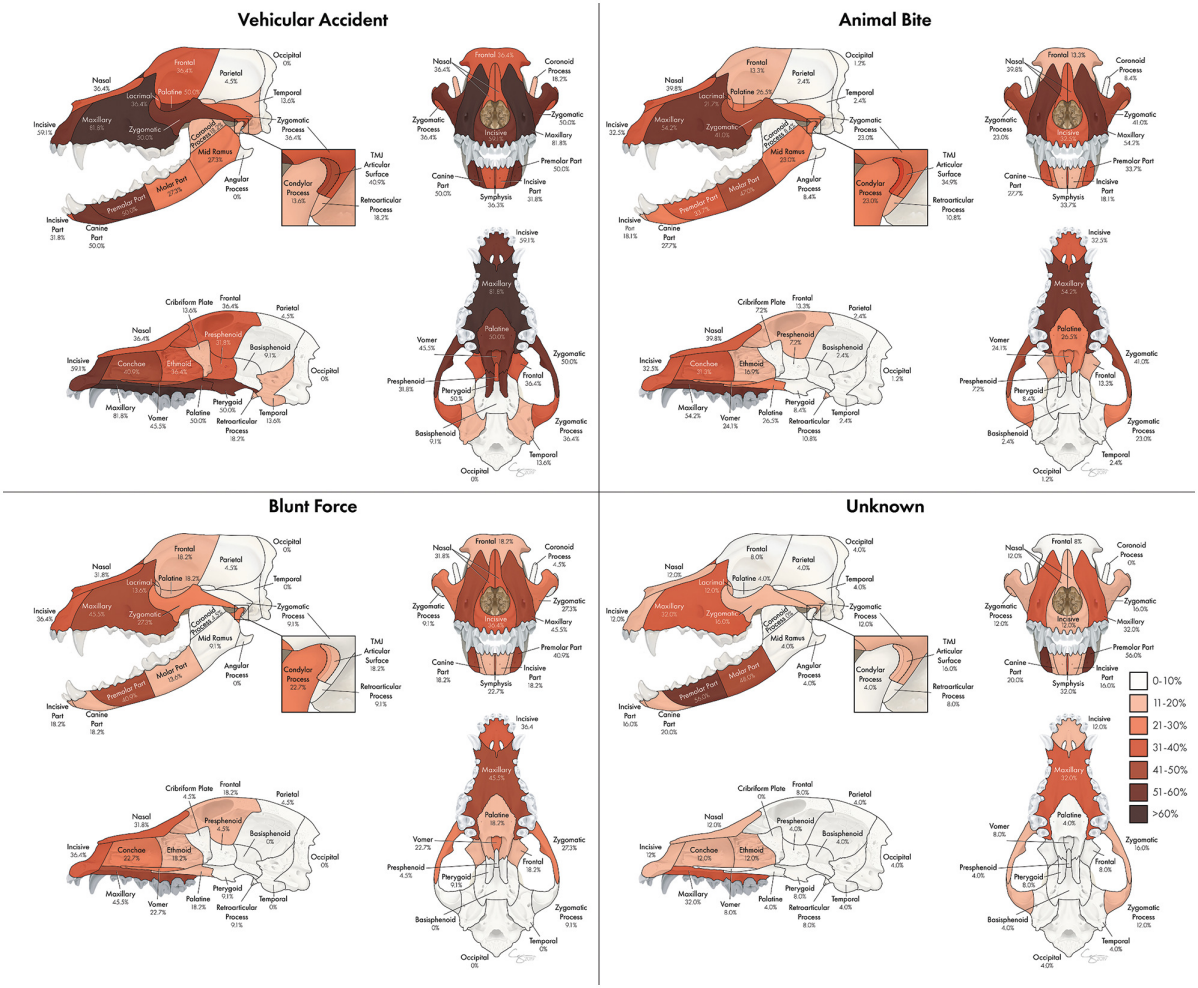
Array of heatmaps according to trauma etiology. Trauma etiologies which accounted for fewer than 20 cases (fall from height, crush injury, ballistic injury) were not included in this portion of the analysis.

The proportion of fractures at each location varied according to etiology as seen in Figure 10. Etiologies that occurred in <20 dogs in the study population included crush injuries, falls from height, and ballistic injuries. These were not included in this part of the assessment. For vehicular accidents, animal bites, and blunt force trauma, the maxillary bone was the most commonly fractured region, occurring in 81.8, 54.2, and 45.5% of cases, respectively. However, in cases of CMF trauma occurring secondary to an unknown etiology, the premolar part of the mandible was the most likely to be fractured, occurring in 56.0% of cases. In cases of vehicular trauma, the premolar and canine parts of the mandible were fractured in 50% of patients, whereas the molar part was more likely (47.0%) to be fractured in animal bites and the premolar part (41.9%) in blunt force traumas. Fractures of the TMJ articular surface also exhibited variation according to trauma etiology such that animal bites and vehicular accidents resulted in fractures in 34.9 and 40.9% of cases, whereas blunt force trauma and unknown trauma only resulted in articular surface fractures in 18.2 and 16.0% of patients, respectively. The frontal (36.4%) and temporal bones (13.6%) were most likely to be fractured in cases of vehicular trauma. The occipital and parietal bones were infrequently fractured in all trauma etiologies.

### Demographic Data and Trauma Etiology

A Fisher's exact test revealed no significant association between trauma etiology and sex (*p* = 0.29). Similarly, a Kruskal-Wallis equality-of-populations rank test revealed no significant difference in patient age between trauma etiologies (*p* = 0.34). However, a Pearson chi-squared revealed that there were significant (*p* < 0.001) associations between patient size and trauma etiology as seen in Table 2. Specifically, patients < 10 kg were significantly less likely to be affected by vehicular trauma. Patients between 20 and 40 kg were significantly more likely to be affected by vehicular trauma and less likely to be affected by an animal bite. Patients > 40 kg were significantly more likely to have experienced blunt force trauma.

**Table 2:** Chi-squared analysis of patient size and trauma etiology.

		**Patient size**					
		** <10 kg**	**10–20 kg**	**20–40 kg**	**>40 kg**	**Juvenile**	**Total**
Crush/slow
	Observed	0	0	2	0	1	3
	Expected	1.3	0.3	0.6	0.1	0.8	3
	Chi-square	1.3	0.3	3.7	0.1	0.1	5.4
Vehicular
	Observed	2	5	9	2	4	22
	Expected	9.2	2.1	4.1	0.9	5.6	22
	Chi-square	5.6*	3.9	5.7*	1.2	0.5	16.9
Animal bite
	Observed	42	3	3	2	33	82
	Expected	34.7	8	15.6	3.5	21.1	83
	Chi-square	1.5	3.2	10.2*	0.7	6.7	22.2
Fall from height
	Observed	2	1	1	0	0	4
	Expected	1.7	0.4	0.8	0.2	1	4
	Chi-square	0.1	1	0.1	0.2	1	2.3
Ballistic
	Observed	1	0	5	0	0	6
	Expected	2.5	0.6	1.1	0.3	1.5	6
	Chi-square	0.9	0.6	13.3	0.3	0.15	16.6
Blunt force
	Observed	6	4	7	3	2	22
	Expected	9.2	2.1	4.1	0.9	5.6	22
	Chi-square	1.1	1.6	2	4.6*	2.3	11.6
Other/unknown
	Observed	16	3	4	0	2	25
	Expected	10.5	2.4	4.7	1.1	6.4	25
	Chi-square	2.9	0.1	0.1	1.1	3	7.2
	Total	69	16	31	7	42	165

Asterisks (*) indicate significant differences between expected and observed incidence of cases.

### Demographic Data and Fracture Location

Sex: There was a significant difference in presence or absence of lacrimal bone (*p* = 0.044) and conchae (*p* = 0.010) fractures according to sex such that intact animals (groups 3 and 4) were more likely to have fractured the lacrimal bone (30–36%) than were neutered animals (13–15%) and more likely to have fractured the conchae (35–44%) than neutered animals (13–20%). In addition, intact females (group 3) were significantly (*p* = 0.007) more likely (54%) to have fractured the nasal bone than other sex groups (21–30%).

Age: When there was a significant association between age and fracture location, younger animals were consistently more likely to have sustained fractures in all bones/regions than were older animals (*p* < 0.05) with the exception of the premolar part of the mandible which showed that dogs with fractures in this region were significantly (*p* = 0.029) older (5.0 years) than those without fractures in this region (3.8 years).

Size: There were two significant associations between size and presence of fractures at particular locations. There was a significant difference in presence or absence of pterygoid fractures according to size (*p* = 0.044) such that dogs that were <10 kg (group 1) were less likely to have a fracture in this region (8.7%) than were any of the other size groups (25–28.6%). In addition, there was a significant difference in presence or absence of lacrimal bone fractures according to size (*p* = 0.044) such that dogs that weighed 10–20 kg and 20–40 kg (groups 2 and 3) were more likely to have a fracture in this region (37.5 and 25.8%, respectively) than were either of the other size groups (8.7–14.3%).

### Demographic Data and Fracture Morphology

Sex: When there were significant associations between sex and severe displacement of fractures of particular locations, intact animals were significantly (*p* < 0.05) more likely than neutered animals to have sustained severe displacement of fractures of the conchae, lacrimal, and palatine bones. Intact females were significantly (*p* = 0.008) more likely (21%) to have severe displacement of the premolar part of the mandibles than were other sex groups (0–11%), whereas neutered males were more likely (7.9%) to have severe displacement of the angular process than were other sex groups (0–2.5%; *p* = 0.025).

When there were significant associations between sex and severe fragmentation of fractures at particular locations, intact animals were significantly (*p* < 0.05) more likely than neutered animals to have sustained severe fragmentation of fractures of the conchae, lacrimal, and palatine bones. Intact females were more likely to have severe fragmentation of the condylar process (7.7%; *p* = 0.037) and articular surface of the TMJ (13%; *p* = 0.032) than were other sex groups (0–2.6% and 0–5.3%, respectively). Finally, there was a significant difference in presence or absence of severe fragmentation of the maxilla according to sex (*p* = 0.24) such that spayed females (group 1) were less likely to have severe fragmentation (27%) than were any of the other sex groups (38–51%).

Age: When there was a significant association between age and severe displacement, younger animals were more likely to have sustained severely displaced fractures in particular locations than were older animals (*p* < 0.05) without exception.

When there was a significant association between age and severe fragmentation, younger animals were more likely to have sustained severely fragmented fractures of particular locations than were older animals (*p* < 0.05). The exception was the zygomatic process for which dogs with severely fragmented fractures of this region were significantly (*p* = 0.047) older (4.3 years) than those without severely fragmented fractures (2.4 years).

Size: There were two significant associations between size and severe fragmentation of fractures. There was a significant difference in presence or absence of severely fragmented ramus fractures according to size (*p* = 0.033) such that dogs that were <10 kg or > 40 kg were less likely to have a severely fragmented fracture in this region (0–2.9%) than were any of the other size groups (16–19%). In addition, there was a significant difference in presence or absence of severely fragmented pterygoid fractures according to size (*p* = 0.036) such that dogs that weighed 10–20 kg (group 2) were more likely to have a severely fragmented fracture in this region (19%) than were any of the other size groups (0–6.5%).

There were two significant associations between size and severe displacement of fractures of particular locations. There was a significant difference in presence or absence of severely displaced pterygoid fractures according to size (*p* = 0.026) such that dogs that weighed <10 kg (group 1) were less likely to have a severely displaced fracture in this region (1.4%) than were any of the other size groups (12–14%). In addition, there was a significant difference in presence or absence of severely displaced ethmoid fractures according to size (*p* = 0.033) such that dogs that weighed <10 kg (group 1) or > 40 kg (group 4) were less likely to have a severely displaced fracture in this region (3 and 0%, respectively) than were either of the other size groups (16–19%).

## Discussion

This comprehensive study documents CMF trauma in dogs using CT as a diagnostic tool and provides a detailed description and mapping of fracture location, morphology, and etiology. We report several key findings. First, dog age and sex were not associated with trauma etiology. However, dog size was associated with trauma etiology. Second, although causes of CMF trauma vary, the most common trauma etiology was animal bite. Third, the maxillary bone and the premolar and molar teeth regions of the mandible were the most commonly fractured. In addition, the more exposed the anatomical region, the higher the probability of severe fracture fragmentation and displacement. In addition, we demonstrated that the most commonly fractured regions of the skull vary according to the etiology of the causative trauma. Finally, younger dogs exhibited more severe fragmentation in particular locations as compared to older dogs.

The present study demonstrates that small breed dogs are less likely to suffer CMF trauma as a result of a vehicular accident. This may be explained by several possibilities. First, it is likely that small breed dogs are less likely to be off leash near vehicles and hence, less likely to be involved in a vehicular accident. Another possibility is that dogs with a small head would die immediately following vehicular trauma rather than presenting to our hospital. The reason that medium-large breed dogs are more likely to sustain CMF trauma from vehicular accidents than from animal bites may reflect on their size (i.e., the larger the dog the less likely it is to be on the receiving end of a bite). In addition, blunt force trauma affected a number of giant breed (> 40 kg) dogs. This could be reflective of the fact that when they do collide with an object, there is often greater momentum involved.

Overall, the most common trauma etiology in our study involved an animal bite, whereas the previous literature has not consistently demonstrated a predominant etiology. For example, several studies (20, 21) found that vehicular accidents accounted for the majority (53–100%) of mandibular fractures, whereas others have found that an animal bite was the most common etiology (19, 22–24). It has also been found that dogs are less likely to sustain CMF trauma after a vehicular accident than they are to sustain trauma to other body parts, possibly due to an instinct to turn the head away from an oncoming object (24). In our study, unknown trauma and vehicular trauma were less common than animal bites. Although this discrepancy between studies may be partially due to regional or temporal variations, as has been pointed out in other publications (24), it is also possible that inconsistent reporting, lack of historical access to CT, and small sample sizes have previously prevented recognition of the most common causes of CMF trauma.

The most commonly fractured region was the maxillary bone followed by the premolar and molar parts of the mandibles. Our findings are in agreement with previous reports on fractured regions of the mandibles. However, our findings differ with regards to incidence of fracture in other regions. Previous reports, which did not all utilize CT for diagnosis, demonstrated that the mandible is vastly more likely to be fractured than other parts of the skull (19, 22, 25). Specifically, the premolar-molar part of the mandibles have been reported to be the most commonly fractured (21) and this may be dependent on patient size (23). In contrast, our study found that the maxillary bone is slightly more likely than the premolar-molar teeth region of the mandibles to be fractured. Previous reports on the physical location of CMF fractures due to trauma are sparse and typically focused on the mandibles. Historically, access to CT was much more limited, so concentrating on mandibular fractures likely reflects the relative ease of interpreting skull radiographic images of the mandible as compared to the difficulty of interpreting the superimposed structures of the maxilla, skull base, and cranial vault (29). This is important given that standard skull radiographs have been documented to significantly underdiagnose the number of fractures in a CMF trauma patient as compared to computed tomography (12). It is not surprising that the maxillary bone and premolar/molar parts of the mandibles are the most commonly fractured regions. As has been described elsewhere (1, 3), the maxilla of the dog is, in many breeds, a prominent and exposed structure and is therefore more susceptible to traumatic insults than other craniomaxillofacial structures. The premolar/molar part of the mandible is similarly exposed to traumatic insults, especially those occurring from the side as opposed to frontally.

Fragmentation and displacement of fractures tended to be more severe at the most exposed regions of the skull which follows the rationale described above. Furthermore, younger dogs exhibited more severe fragmentation as compared to older dogs. The maxillary region, in general, is composed of thin, lightweight bones interposed with nasal and paranasal passages (1, 3). As may be expected in such an area, when fractured, these regions experience more severe impact and therefore exhibit more displacement and fragmentation than other, more protected structures such as the skull base. It is important to note, however, that the low overall incidence of skull base and cranial vault fractures in our study may also reflect the likelihood that such injuries are more often rapidly fatal and, therefore, these patients may not have lived long enough to enter our patient population. With regards to age and fragmentation, it is plausible that the younger the dog, the more fragile are the CMF bones and, in addition to the presence of cranial sutures, predispose to excessive fragmentation following CMF trauma.

The most commonly fractured location varied according to trauma etiology. Specifically, vehicular accidents tended to result in more regions with a higher probability of being fractured than other trauma types, likely due to the velocity and impact with which vehicles strike animals. Notably, the pterygoid bones were fractured in vehicular accidents more often than in other types of trauma. This is essential for clinicians to understand as these injuries can be easy to miss and can greatly affect patient discomfort and ability to swallow. Animal bites are also likely to result in multiple areas with a relatively high probability of fracture, but tend to be centered mostly on the maxillary bone, zygomatic bone, and molar part of the mandibles. One explanation is that if a dog is bitten with the upper teeth grasping the muzzle and the lower teeth grasping either the inside of the oral cavity or below the mandible, these areas of fracture are logical. The palatine and frontal bones were similarly affected in cases of blunt force trauma and animal bites, but were more common in vehicular accidents and rare in unknown trauma types.

The pattern of TMJ fractures is important to note as articular surface fractures were most commonly confined to either the condylar process or the zygomatic process for blunt force and unknown trauma, respectively, but more commonly occurred on both surfaces in vehicular accidents and animal bites. Thorough evaluation of the TMJ following CMF trauma is essential as fractures associated with the articular surface may have long term adverse consequences such as joint pain, reduced mandibular opening, degenerative joint disease, masticatory dysfunction or ankylosis (30, 31). In addition, if the fracture of the TMJ occurred at an early age, it is likely to affect the growth and development of the mandibles (32).

The limitation of this study is inherent to its retrospective design. In addition, the patients included in this study were assessed at a tertiary referral institution, which could have affected the types of CMF trauma included in the study. For example, very mild cases may not have been referred to our institution if the primary veterinarian felt capable of treating the patient. Likewise, very severe cases may have died or been euthanized prior to referral. Because several of the trauma etiologies (crush injuries, fall from height, and ballistic traumas) occurred infrequently, the sample size for those etiologies was too small to draw any conclusions from the associated data. Finally, the inclusion of unknown trauma etiology category can be viewed as a limitation as it does not reveal precise information.

In conclusion, by assessing CT images of the entire CMF region in a large population of patients, this study has highlighted the most commonly fractured regions of the skull as well as the most common causative traumatic insults. In addition, we provided basic information regarding trauma etiology and the regions of the skull that are most likely to be fractured. In turn, this allows veterinarians to focus their physical exams and diagnostic imaging in the appropriate regions. Importantly, a major takeaway from this study is that every bone of the CMF region was fractured in at least one case and many cases had a large number of fractured regions. Therefore, the need for careful assessment of the entire CMF region using CT has been underscored. In part 2, we report on the specific fracture locations and their tendency to co-fracture with other locations, as well as further elucidating the relationships between trauma etiology, fracture morphology, and fracture location.

## Data Availability Statement

The datasets generated for this study are available on request to the corresponding author.

## Ethics Statement

Ethical review and approval was not required for the animal study because the study is retrospective in nature and included clinical cases, hence, it is exempt from IACUC requirements. Written informed consent for participation was not obtained from the owners because the study is retrospective in nature and, hence, it is exempt from written informed consent.

## Author Contributions

MD: Study concept and design, image analysis, data acquisition, analysis and interpretation, drafting of the manuscript, final approval of the version to be published. RP: Study concept and design, image analysis, drafting of the manuscript, final approval of the version to be published. BA and FV: Study concept and design, data interpretation, drafting of the manuscript, final approval of the version to be published. PK: Data analysis and interpretation, drafting of the manuscript, final approval of the version to be published.

## Conflict of Interest

The authors declare that the research was conducted in the absence of any commercial or financial relationships that could be construed as a potential conflict of interest.
