# Corrigendum: Craniomaxillofacial Trauma in Dogs—Part I: Fracture Location, Morphology and Etiology

**DOI:** 10.3389/fvets.2022.882505

**Published:** 2022-03-16

**Authors:** Mercedes H. De Paolo, Boaz Arzi, Rachel E. Pollard, Philip H. Kass, Frank J. M. Verstraete

**Affiliations:** ^1^School of Veterinary Medicine, William R. Pritchard Veterinary Medical Teaching Hospital, University of California, Davis, Davis, CA, United States; ^2^Department of Surgical and Radiological Sciences, University of California, Davis, Davis, CA, United States; ^3^Department of Population Health and Reproduction, School of Veterinary Medicine, University of California, Davis, Davis, CA, United States

**Keywords:** craniomaxillofacial, trauma, computed tomography, fracture, displacement, dog

In the original article, there was an error in the text. The sentence stating “Patients <40 kg were significantly more likely to have experienced blunt force trauma” should have read “Patients > 40 kg were significantly more likely to have experienced blunt force trauma.” This typographical error was not reflected elsewhere in the tables or conclusions of the manuscript.

A correction has been made to *Results, Demographic Data and Trauma Etiology, Paragraph 1*. The corrected paragraph is shown below.

A Fisher's exact test revealed no significant association between trauma etiology and sex (*p* = 0.29). Similarly, a Kruskal-Wallis equality-of-populations rank test revealed no significant difference in patient age between trauma etiologies (*p* = 0.34). However, a Pearson chi-squared revealed that there were significant (*p* < 0.001) associations between patient size and trauma etiology as seen in **Table 2**. Specifically, patients < 10 kg were significantly less likely to be affected by vehicular trauma. Patients between 20 and 40 kg were significantly more likely to be affected by vehicular trauma and less likely to be affected by an animal bite. Patients > 40 kg were significantly more likely to have experienced blunt force trauma.

The authors apologize for this error and state that this does not change the scientific conclusions of the article in any way. The original article has been updated.

## Publisher's Note

All claims expressed in this article are solely those of the authors and do not necessarily represent those of their affiliated organizations, or those of the publisher, the editors and the reviewers. Any product that may be evaluated in this article, or claim that may be made by its manufacturer, is not guaranteed or endorsed by the publisher.

